# Prevalence and correlates of digital violence among female members of the faculty of medicine, Alexandria University

**DOI:** 10.1186/s12889-026-26514-1

**Published:** 2026-04-01

**Authors:** Aida Mohey Mohamed

**Affiliations:** Community Medicine & Public Health, Faculty of Medicine, Alexandria University, Alexandria City, Egypt

**Keywords:** Digital violence, Gender-based violence, Cyber ethics, Digital safety awareness, Reporting barriers, Higher education, Egypt

## Abstract

**Background:**

Digital violence has emerged as a significant extension of gender-based violence within academic contexts, where technological tools enable novel forms of harassment, surveillance, and online exploitation. Women in higher-education institutions particularly within medical faculties, face heightened vulnerability due to structural hierarchies, public digital visibility, and persistent gender inequities. Despite global recognition of this issue, evidence from low- and middle-income countries remains limited.

**Objective:**

This study sought to determine the prevalence, correlates, and institutional determinants of digital violence among female members of the Faculty of Medicine, Alexandria University, while assessing their digital safety awareness, perceptions of cyber ethics, and barriers to reporting such incidents.

**Methods:**

A cross-sectional descriptive study was conducted from April to June 2025 among 420 female participants, including undergraduate students, teaching assistants, and faculty staff members. A structured pretested self-administered questionnaire measured digital violence experience, digital safety awareness (DSA), cyber-ethics perception (CEP), reporting knowledge (RK), barriers (BTR), facilitators to reporting (FTR), help-seeking preferences, training needs and institutional climate. Data were analyzed using SPSS v26 through descriptive statistics, correlations, ANOVA, and multivariate logistic regression. Open-ended responses were thematically analyzed to complement quantitative findings.

**Results:**

Overall, 40% of participants reported experiencing digital violence, primarily in the forms of online harassment (22.6%), hate speech (17.1%), and cyberstalking (14.8%). The most common consequences included psychological distress (66.7%) and impaired academic or work performance (42.3%). Higher DSA, CEP, RK, and FTR scores were associated with lower odds of digital violence, whereas greater perceived barriers (BTR) significantly increased risk (Adjusted OR = 1.60; *p* < 0.001). Being an undergraduate, single, or a heavy internet user (> 5 h/day) increased vulnerability, while prior digital-safety training was protective.

**Conclusion:**

Digital violence poses a growing threat to female academics and students in medical education. Addressing it requires stronger institutional support systems, expanded digital-safety education, and the establishment of confidential, anonymous reporting mechanisms.

**Supplementary Information:**

The online version contains supplementary material available at 10.1186/s12889-026-26514-1.

## Introduction

Violence against women remains a critical global issue encompassing public health, human rights, and institutional dimensions. With the widespread digitalization of society, new forms of technology-facilitated abuse, often termed cyber-violence or digital gender-based violence, have emerged, mirroring and intensifying existing offline power imbalances. Within academic environments, this form of violence can appear as online harassment, stalking, professional or social media abuse, and the non-consensual distribution of personal images. However, evidence on its prevalence among academic personnel especially female faculty in medical institutions remains limited. According to the European Institute for Gender Equality (EIGE) (2025), advancements such as artificial intelligence have increased women’s vulnerability to online abuse, while feminist research underscores that such digital violence is rooted in entrenched gender disparities and institutional power structures that are still inadequately addressed in higher education contexts [1].

Extensive research has explored cyberbullying and digital violence among school and university students, yet far less attention has been given to academic staff. Notar et al. (2013) [2] examined cyberbullying in secondary education, outlining participant roles, prevalence, and gender variations. Watts (2017) [3] emphasized that although most research centers on students, awareness of online aggression directed toward teachers and faculty members is increasing. Evidence indicates that digital harassment within academic workplaces is a genuine concern. Cassidy et al. (2014) [4] reported that 17% of university faculty in a Canadian study had experienced cyberbullying, underscoring the influence of power dynamics and institutional environments. Furthermore, Güneş (2022) [5] showed that women are disproportionately subjected to online harassment, stalking, and image-based abuse, reflecting a wider continuum of gender-based violence. The European Institute for Gender Equality (EIGE) (2025) [1] also confirmed that women and girls are significantly more likely than men to face cyber-violence, while Yarbrough et al. (2023) [6] highlighted faculty perceptions of cyberbullying and emphasized the importance of institutional factors in understanding and addressing these incidents.

Digital violence is shaped by a complex interplay of individual, behavioral, and institutional factors that influence both susceptibility and response. Key correlates include digital safety awareness, perception of cyber ethics, reporting knowledge, and perceived barriers or facilitators to reporting [7, 8]. To guide this research, the study adopts the multi-level theoretical framework proposed by the UniSAFE project [9], which conceptualizes gender-based violence within research organizations across three interconnected levels: micro (individual), meso (organizational), and macro (societal). This framework underscores how structural power relations, gender inequality, and institutional responses interact to perpetuate or mitigate digital violence. In this context, individual-level factors encompass personal demographics, digital safety awareness, and familiarity with reporting mechanisms; behavioral-level factors relate to digital practices and understanding of institutional policies; and institutional-level factors involve organizational culture, the availability of digital safety training, and the effectiveness of reporting and support systems [10–12].

The Faculty of Medicine at Alexandria University offers a unique setting to study digital violence among female academics. Medical faculties are high‑stakes environments with hierarchical structures and frequent digital communication. Female faculty members may face gendered expectations and digital exposure risks, yet research in low-moderate income countries (LMICs) like Egypt is scarce. This study fills critical gaps by exploring prevalence, associated factors, and institutional correlates. It aims to generate data that inform university policies, enhance digital‑safety education, and promote gender equity. The objective is to determine the prevalence of digital violence in the academic environment among female members of the Faculty of Medicine at Alexandria University and identify the associated individual, behavioral and institutional factors (correlates) linked to its occurrence and reporting.

## Methodology

### Study design

This study adopted a convergent mixed-methods design, where data of a quantitative descriptive cross-sectional survey were complemented by qualitative thematic analysis of open-ended responses. Integration occurred at the interpretation stage, allowing qualitative findings to contextualize quantitative results (Good Reporting of A Mixed Methods Study (GRAMMS)-guided).

### Study setting and duration

The study was conducted at the Faculty of Medicine, Alexandria University, Egypt. According to official faculty records for 2024, the faculty included approximately [7,200] undergraduate students, of whom [62.3] % were females, and 450 academic staff members and 600 teaching assistants, with females constituting [58.4] % of the total academic and assistant teaching staff.

Data collection was carried out between April and June 2025, following approval from the Faculty Ethics Committee and after obtaining informed consent from all participants.

### Study population and eligibility criteria

The study targeted female members affiliated with the Faculty of Medicine, Alexandria University, including:

- Undergraduate students in the national program across all academic years.
- Academic faculty staff members and teaching assistants from various departments.

Inclusion criteria:

- Female participants aged 18 years or older.
- Currently enrolled as students or employed as academic staff members or teaching assistants at the Faculty of Medicine, Alexandria University.
- Provided informed consent to participate in the study.

Exclusion criteria:

- Incomplete, inconsistent, or duplicate questionnaire responses.

### Sample size and sampling technique

The study estimated the 12-month prevalence of any digital violence (binary outcome) among female members of the faculty of medicine using stratified random sampling (students, assistant teaching & academic staff, faculty affairs). The core sample size follows Cochran’s formula for a proportion [13, 14] with 95% confidence, conservative *p* = 0.5, and overall precision ± 5%: $\:{n}_{0}=\frac{{z}^{2} p(1-p)}{{d}^{2}}=\frac{{1.96}^{2}\times\:0.5\times\:0.5}{{0.05}^{2}}\approx\:384$. Applying the finite population correction (FPC) to the total frame $\:N=\mathrm{8,250}$ gives about 367, and with a design effect (≈ 1.1) plus 20% inflation for nonresponse, the statistically inflated sample size was estimated at approximately 449 participants, however, data collection was concluded at 420 completed responses due to logistical feasibility and acceptable statistical power.

Within each stratum, a simple random sample is drawn from clean female rosters (students by year, teaching & academic staff faculty by department/unit), with an oversample reserve list (~ 30%) to replace non-contacts in order, and a contact protocol (multichannel, up to 3 attempts, short survey, confidentiality wording) to keep nonresponse ≤ 20%. Base analysis weights are the inverse of selection probability, i.e. $\:{w}_{h}={N}_{h}/{n}_{h}$. These base weights can be further adjusted for differential nonresponse inside each stratum and post-stratified (raked) to known margins (e.g. student year, faculty department) so that weighted estimates of digital violence remain unbiased and comparable across groups. (supplementary file1)

Data Collection Instrument.

Data were collected using a structured, self-administered questionnaire developed by the researcher for this study (supplementary file2). The instrument was designed in English based on an extensive literature review of digital violence, digital safety and online ethics among university populations [14–19]. It was validated by three domain experts (public health, medical education, and digital safety) and pretested on 30 participants for clarity and reliability (Cronbach’s α values > 0.70 for all main scales). The questionnaire was administered in English, which is the official language of instruction at the Faculty of Medicine, Alexandria University. All reported experiences of digital violence refer to incidents occurring within the previous 12 months.

Responses for most items were measured on a 5-point Likert scale ranging from *1 (Strongly Disagree)* to *5 (Strongly Agree)*. Reverse-coded items were recalculated accordingly.

The questionnaire comprised eight structured sections and one open-ended section:

1. Demographics – 7 items on age, role, and other background factors.
2. Digital Habits & Exposure – 5 behavioral questions.
3. Digital Safety Awareness (DSA) – 15 items assessing knowledge and practices.
4. Cyber-Ethics Perception (CEP) – 12 items evaluating ethical attitudes.
5. Experience of Digital Violence (EDV) & reporting – multiple-choice questions on exposure types, impacts and reporting (10 items).
6. Reporting Knowledge (RK), Barriers (BTR) & facilitators to reporting (FTR) – 17 items measuring familiarity with procedures and perceived obstacles & facilitators.
7. Help-Seeking Preferences – 3 items on reporting channels and support.
8. Institutional Climate (IC) – 5 items on the perceived culture of online respect.
9. Training Needs – 3 items regarding preferred training formats and content.
10. Open-ended items – for qualitative suggestions to improve reporting and safety (I1–I3).

Composite Reliability (CR) values and standardized factor loadings were calculated and are presented in Supplementary File 3 to further support scale validity and facilitate future cross-population comparisons.

Composite scores were computed for each major construct. The indices Digital Safety Awareness (DSA), Reporting Knowledge (RK), and Institutional Climate (IC) used identical categorization thresholds: mean scores below 3.00 were classified as *low*, scores between 3.00 and 3.99 as *moderate*, and scores ≥ 4.00 as *high*. In contrast, Cyber-Ethics Perception (CEP) had slightly higher cutoffs, where < 3.20 indicated *low*, 3.20–4.19 represented *moderate*, and ≥ 4.20 denoted *high* perception. Finally, Barriers to Reporting (BTI) was interpreted in the opposite direction, as lower values reflected fewer obstacles. Scores ≤ 2.59 were considered *low* (few barriers), 2.60–3.59 as *moderate*, and ≥ 3.60 as *high* (many barriers).

### Data collection procedure

The survey form was administered both electronically (Google Forms) and as paper-based copies distributed in lecture rooms, and faculty offices. Participation was voluntary and anonymous, and participants were allowed to skip any question they felt uncomfortable answering. An informed consent section preceded the questionnaire, explaining purpose, confidentiality, and the right to withdraw at any time.

### Data analysis

Data were analyzed using the Statistical Package for the Social Sciences (SPSS) version 26. Descriptive statistics including frequencies, percentages, means, and standard deviations were computed to summarize participants’ demographic characteristics, digital practices, and experiences of digital violence. Composite scores for the main constructs, Digital Safety Awareness (DSA), Cyber-Ethics Perception (CEP), Reporting Knowledge (RK), Barriers to Reporting (BTR), Facilitators of Reporting (FR), and Institutional Climate (IC) were generated from Likert-scale items, and internal consistency was assessed using Cronbach’s α reliability coefficients. Cronbach’s α was computed to test internal consistency (target α ≥ 0.70) [20]. All scales show good internal consistency, with Cronbach’s α values ranging from 0.78 to 0.86, exceeding the commonly accepted threshold of 0.70. This indicates that the items within each scale are reliably measuring consistent underlying constructs.

Inferential analyses were applied to test associations between digital violence experience and individual or institutional factors. Independent-sample *t*-tests and one-way ANOVA were used to compare mean scale scores across participant subgroups, with post-hoc Tukey tests for pairwise differences. Spearman’s correlation assessed inter-relationships among continuous variables. Multicollinearity was assessed using Variance Inflation Factors (VIF), all of which were below 2.5, indicating no significant multicollinearity.

A multivariate binary logistic regression model was then constructed to identify independent predictors of digital violence, reporting adjusted odds ratios (AOR) with 95% confidence intervals. Model adequacy was evaluated using the Hosmer–Lemeshow goodness-of-fit test and Nagelkerke R². Significance was set at *p* < 0.05.

Qualitative Analysis: Open-ended responses were analyzed using thematic analysis. Two researchers independently coded responses, resolved discrepancies by consensus, and derived final themes. Representative quotes were selected to illustrate dominant themes.

## Results

A total of 420 participants were included in the study. Table 1. presents demographic and digital-behavioral profile of the study participants. The mean age of 26.4 ± 5.7 years indicates a relatively young population, with a median of 24 years and an age range extending from 18 to 55 years. Most respondents were undergraduate students (66.7%), followed by teaching assistants (19.0%) and academic faculty members (14.3%), showing that students formed the majority of the sample.

**Table 1:** Sociodemographic and digital characteristics of female members of the faculty of medicine (*n* = 420)

Variable	Statistic/Category	Frequency (*n*)	%
Age (years)	Mean ± SD	26.4 ± 5.7	
	Median (IQR)	24 (22–30)	
	Minimum – Maximum	18–55	
Academic role	Undergraduate students	280	66.7
	Teaching assistants	80	19.0
	Academic faculty staff	60	14.3
Marital status	Single	310	73.8
	Married	100	23.8
	Divorced/Widowed	10	2.4
Average daily internet use	< 2 h	40	9.5
	2–5 h	180	42.9
	> 5 h	200	47.6
Primary digital platforms used#	WhatsApp	360	85.7
	Facebook	340	81.0
	Instagram	280	66.7
	University portals	240	57.1
	Telegram	120	28.6
	Others (TikTok, messenger)	60	14.3
Primary devices used #	Phone	403	96.0
	Laptop	294	70.0
	Tablet	101	24.0
	Shared / Clinic PC	76	18.0
Public academic/professional account	Yes	185	44.0
	No	235	56.0
Unwanted contact from patients/public online	Yes	84	20.0
	No	336	80.0

^#^Allow multiple responses; percentages calculated from total *n* = 420

In terms of marital status, the majority were single (73.8%), while 23.8% were married and 2.4% divorced or widowed. Regarding internet engagement, nearly half reported using the internet more than 5 h daily (47.6%), and 42.9% used it for 2–5 h, confirming heavy online activity among participants.

With respect to digital platforms, WhatsApp (85.7%) and Facebook (81.0%) were the most commonly used, followed by Instagram (66.7%) and university portals (57.1%), while Telegram (28.6%) and other applications such as TikTok and Messenger (14.3%) were less prevalent.

When examining devices, the mobile phone (96.0%) dominated as the primary access tool, with considerable use of laptops (70.0%), and lower proportions using tablets (24.0%) or shared/clinic computers (18.0%).

Concerning online professional presence, 44.0% reported having a public academic or professional account, compared to 56.0% without. A fifth of participants (20.0%) reported receiving unwanted online contact from patients or the public, while 80.0% did not encounter such incidents.

Table 2. shows experience of digital violence (EDV) and perceived impacts among female participants. Almost 40% reported experiencing digital violence, while 60% had not. This indicates that a notable proportion of respondents have been exposed to some form of online aggression or harmful digital behavior within their digital environments.

**Table 2:** Experience of digital violence (EDV) among female members of the faculty of medicine (*n* = 420)

Variable	Category	Frequency (*n*)	*Percentage (%)
Overall Experience of Digital Violence	Yes	168	40.0
	No	252	60.0
Types of Digital Violence Experienced#	Harassing or threatening messages/comments	95	22.6
	Cyberstalking (repeated unwanted online contact/monitoring)	62	14.8
	Non-consensual sharing of images (including intimate)	41	9.8
	Impersonation/fake accounts in my name	56	13.3
	Doxxing (exposure of private data, e.g., phone, address)	38	9.0
	Blackmail/extortion (including sexual extortion “sextortion”)	47	11.2
	Online gender-based hate speech	72	17.1
	Distribution of manipulated/deepfake media	33	7.9
Frequency of Incidents (among victims)	Once	44	26.2
	Occasionally (2–3 times/year)	79	47.0
	Frequently (≥ monthly)	45	26.8
Perceived Impacts#	Psychological distress (anxiety, fear)	112	66.7
	Academic/work performance affected	71	42.3
	Avoidance of online activities	64	38.1
	No significant impact	26	15.5

^#^Multiple responses allowed for types and impacts; categories are not mutually exclusive

The study participants experienced a wide range of digital violence forms. The most frequently reported types of digital violence were harassing or threatening messages or comments (22.6%), followed by online gender-based hate speech (17.1%) and cyberstalking through repeated unwanted contact or monitoring (14.8%). Other reported forms included impersonation or fake accounts created in the respondent’s name (13.3%), blackmail or extortion including sexual extortion (11.2%), non-consensual sharing of images (9.8%), doxxing or exposure of private information such as phone numbers or addresses (9.0%), and distribution of manipulated or deepfake media (7.9%).

Regarding the frequency of incidents among victims, 26.2% reported being targeted once, while 47.0% experienced such incidents occasionally (two to three times per year). However, 26.8%, indicated that they were subjected to digital violence frequently, on a monthly or more regular basis.

The perceived impacts of these experiences were mainly psychological and academic. About two-thirds (66.7%) of victims reported psychological distress such as anxiety or fear. Additionally, 42.3% indicated that their academic or work performance was affected, and 38.1% reported avoidance of online activities as a consequence. Only 15.5% of respondents stated that the incidents had no significant impact on them.

Table 3. summarizes the mean scores, and distribution levels across five main scales assessing digital safety awareness, ethical perception, reporting behavior, and institutional support. The mean score of Digital Safety Awareness (DSA) was 3.95 ± 0.49, indicating a moderate to high level of awareness. The distribution (10% low, 55% moderate, 35% high) shows that nearly 90% of participants possess at least a moderate understanding of digital safety concepts. Cyber-Ethics Perception (CEP): Exhibits the highest overall mean (4.12 ± 0.40) and a “High” level of perception. More than half (55%) scored high, reflecting strong ethical sensitivity toward responsible digital behavior. Reporting Knowledge (RK): Moderate mean score (3.51 ± 0.49), with most participants (60%) showing moderate familiarity with reporting procedures. Barriers to Reporting (BTI): Mean: 2.90 ± 0.59 indicates a moderate level of perceived barriers, with 30% reporting high obstacles. This suggests that although awareness exists, reporting digital violence remains hindered. The *Facilitators of Reporting (FR)* scale shows a mean score of 3.70 ± 0.52 which indicates a moderate–high overall level among participants. In terms of distribution, more than half of the participants (58%) demonstrated a moderate perception of facilitators that support reporting behaviors. Institutional Climate (IC): Mean score was 2.90 ± 0.51, suggesting a generally less institutional environment. Only (50%) perceive the climate as moderately supportive.

**Table 3:** Mean Scores, and levels of digital safety constructs among female members of the faculty of medicine (*n* = 420)

Scale	Cronbach’s α	Mean ± SD	Overall Level	Low (%)	Moderate (%)	High (%)
Digital Safety Awareness (DSA)	0.86	3.95 ± 0.49	Moderate–High	10	55	35
Cyber-Ethics Perception (CEP)	0.84	4.12 ± 0.40	High	8	37	55
Reporting Knowledge (RK)	0.82	3.51 ± 0.49	Moderate	20	60	20
Barriers to Reporting (BTR)	0.78	2.90 ± 0.59	Moderate	30	50	20
Facilitators of Reporting (FR)	0.81	3.70 ± 0.52	Moderate–High	12	58	30
Institutional Climate (IC)	0.80	2.90 ± 0.51	Low	35	50	15

Table 4 presents the comparison of Digital Safety Awareness (DSA) scores among female members according to their academic role. The mean DSA score for undergraduate students is 3.90 ± 0.45, indicating moderate awareness levels within this group. Teaching assistants show a slightly higher mean score of 3.98 ± 0.52, suggests more consistent awareness levels compared to undergraduates. The academic faculty staff exhibit the highest mean score of 4.12 ± 0.48, reflecting generally higher awareness compared to the other two groups.

**Table 4:** Comparison of digital safety awareness scores of female members of the faculty of medicine (*n* = 420) by academic role and digital violence experience

Variable	Category	*n*	Mean ± SD	Statistical test	*p*-Value	Effect Size
Academic Role	Undergraduate students (UG)	280	3.90 ± 0.45	F = 4.62	0.011*	η² = 0.022
	Teaching assistants (TA)	80	3.98 ± 0.52			
	Academic Faculty staff (AS)	60	4.12 ± 0.48			
	Post-hoc (Tukey):					
	UG vs. AS: *p* = 0.009*					
	TA vs. AS: *p* = 0.211					
	UG vs. TA: *p* = 0.372					
Experience of Digital Violence (EDV)	Yes	168	3.82 ± 0.51	*t* = 3.21	0.001*	Cohen’s *d* = 0.32
	No	252	4.03 ± 0.47			

*Statistically significant at 0.05 level

The mean awareness levels are not identical across groups. A one-way ANOVA test reveals a statistically significant difference in DSA scores among the three academic roles (F = 4.62, *p* = 0.011). The effect size, represented by η² = 0.022, denotes a small but notable difference in DSA scores attributable to academic role variation.

Post-hoc Tukey comparisons further clarify the pairwise differences. The difference between undergraduate students and academic faculty staff is statistically significant (*p* = 0.009), showing that faculty members possess higher awareness scores. However, the differences between teaching assistants and staff (*p* = 0.211) and between undergraduates and teaching assistants (*p* = 0.372) are not statistically significant.

In Table 4, those who had not experienced digital violence reported significantly higher DSA levels (*M = 4.03 ± 0.47*) than those who had (*M = 3.82 ± 0.51*), *t* = 3.21, *p* = 0.001, *d* = 0.32, indicating that greater digital safety awareness is associated with a lower risk of experiencing EDV.

**Fig. 1:**
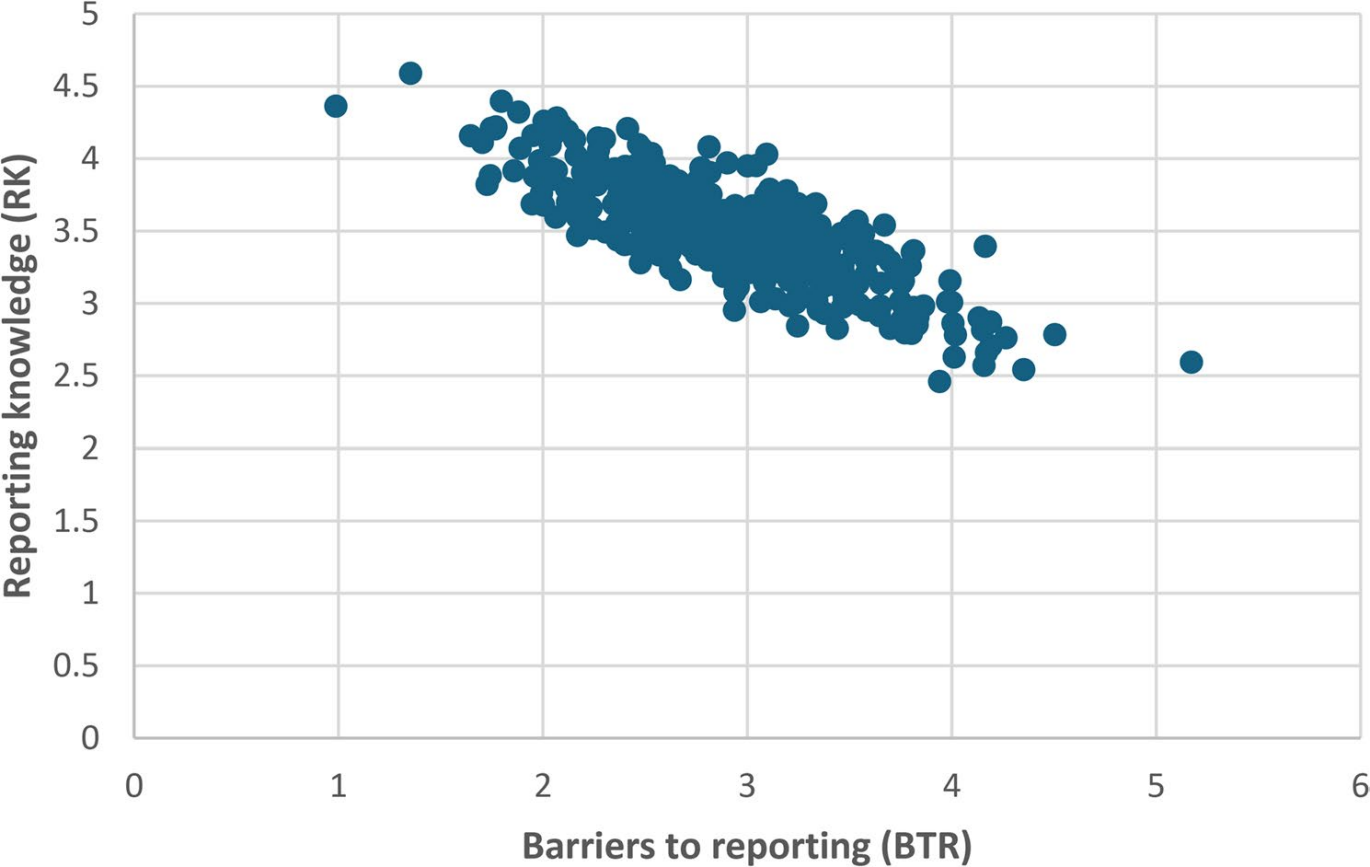
Correlation Between Barriers to Reporting (BTR) and Reporting Knowledge (RK) among Female Members of the Faculty of Medicine (*n* = 420)

The scatter diagram visually (Fig. 1) displays the correlation between Barriers to Reporting (BTI) and Reporting Knowledge (RK) among female members of the Faculty of Medicine. Using the Spearman correlation coefficient (ρ = − 0.52, *p* < 0.001), the analysis confirms a statistically significant negative relationship between BTR and RK, indicating that participants perceiving more barriers tend to have lower knowledge about reporting mechanisms. The correlation strength is moderate.

Table 5 outlines the help-seeking behavior and preferred support mechanisms among the studied female members of the Faculty of Medicine regarding digital violence. Half of the participants (50%) indicated that they would first turn to friends or family if they experienced digital violence. Only a small proportion would approach class advisors or supervisors (15%), and even fewer would seek help from department leadership (6%) or university reporting offices (10%). Mental-health services (7%) and the police (5%) were among the least chosen options.

**Table 5:** Help-seeking and support preferences among female members of the faculty of medicine (*n* = 420)

Variable	Category	*n*	%
First contact if experiencing digital violence	Friend / Family	210	50.0
	Class advisor / Supervisor	63	15.0
	Department leadership	25	6.0
	University reporting office	42	10.0
	Mental-health services	30	7.0
	Police	21	5.0
	Platform tools	29	7.0
Preferred reporting channels (top 3) #	Anonymous web form	231	55.0
	Named web form	76	18.0
	Email	101	24.0
	Phone hotline	59	14.0
	In-person office	88	21.0
	App / Portal	126	30.0
Supports that would help reporting #	Step-by-step guide	189	45.0
	Evidence documentation help	160	38.0
	Confidential advocate	168	40.0
	Status updates on case	126	30.0
	Option to pause or withdraw	97	23.0
	Referral to counseling	118	28.0
	Legal guidance	147	35.0

^#^Multiple selections were allowed per item; therfore totals exceed 100%

When asked about preferred channels for reporting, the majority (55%) favored anonymous web forms, highlighting the importance of privacy and confidentiality. A smaller percentage preferred named web forms (18%) or traditional email contact (24%). Digital and direct communication options like apps/portals (30%) and in-person offices (21%) also appeared moderately preferred, whereas phone hotlines (14%) were less popular.

Regarding supports that could facilitate reporting, nearly half (45%) valued a step-by-step guide. Confidential advocacy (40%) and assistance with evidence documentation (38%) were also frequently chosen. Legal guidance (35%) and regular case status updates (30%) were moderately requested, while referral to counseling (28%) and the option to pause or withdraw (23%) were less emphasized but still notable.

The logistic regression model (Table 6) identifies several significant predictors of experiencing digital violence among the studied female members (*n* = 420). The overall model demonstrates a good fit and explanatory power, with a statistically significant omnibus test (χ² = 92.7, *p* < 0.001) and a Nagelkerke R² of 0.45, indicating that approximately 45% of the variance in digital violence experience is explained by the included variables. Additionally, the Hosmer–Lemeshow goodness-of-fit test (χ² = 5.87, *p* = 0.43) confirms an adequate model fit, suggesting that predicted probabilities align well with observed outcomes.

**Table 6:** Multivariate logistic regression analysis for predictors of digital violence among female members of the faculty of medicine (*n* = 420)

Independent Variables	Adjusted OR (95% CI)	*p*-Value
Age (years)	0.93 (0.86–1.02)	0.095
Academic role (Ref: Faculty staff)		
Undergraduate student	2.23 (1.28–3.88)	0.005 *
Teaching assistant	1.68 (0.90–3.13)	0.094
Marital status (Ref: Married)		
Single	1.91 (1.16–3.15)	0.011*
Divorced/Widowed	1.97 (0.95–4.08)	0.067
Average daily internet use (> 5 h vs. ≤ 5 h)	2.44 (1.55–3.84)	< 0.001*
Previous digital safety training (Yes)	0.56 (0.33–0.95)	0.035*
Digital Safety Awareness (DSA) score	0.68 (0.53–0.87)	0.002*
Cyber-Ethics Perception (CEP) score	0.73 (0.56–0.96)	0.021*
Reporting Knowledge (RK) score	0.77 (0.60–0.99)	0.043*
Barriers to Reporting (BTR) score	1.60 (1.27–2.01)	< 0.001*
Facilitators of Reporting (FR) score	0.72 (0.55–0.94)	0.018*
Institutional Climate (IC) score	0.85 (0.69–1.05)	0.113

Dependent variable: Experience of Digital Violence (Yes/No)

Overall model significance (Omnibus test): χ² = 92.7, *p* < 0.001

Nagelkerke R²: 0.45

Hosmer–Lemeshow test: χ² = 5.87, *p* = 0.43

Age showed a non-significant inverse association with digital violence (*p* = 0.095), indicating a possible trend toward lower risk with increasing age. Regarding academic role, undergraduate students had significantly higher odds of experiencing digital violence (adjusted OR = 2.23, 95% CI: 1.28–3.88, *p* = 0.005) compared to faculty staff, whereas teaching assistants showed a non-significant increase. Single participants also had a significantly higher likelihood of digital violence (adjusted OR = 1.91, 95% CI: 1.16–3.15, *p* = 0.011) compared to married women, while divorced or widowed respondents showed a borderline effect (*p* = 0.067).

Intensive internet use (> 5 h/day) was a strong predictor, doubling the odds of exposure (adjusted OR = 2.44, 95% CI: 1.55–3.84, *p* < 0.001). Conversely, having received prior digital safety training reduced the odds of experiencing digital violence by nearly half (adjusted OR = 0.56, 95% CI: 0.33–0.95, *p* = 0.035).

Among the psychosocial constructs, higher Digital Safety Awareness (DSA), Cyber-Ethics Perception (CEP), Reporting Knowledge (RK), and Facilitators of Reporting (FR) scores were all associated with lower odds of digital violence, each showing statistically significant protective effects. In contrast, higher Barriers to Reporting (BTR) significantly increased the likelihood of being a victim (adjusted OR = 1.60, 95% CI: 1.27–2.01, *p* < 0.001). Institutional Climate (IC) was not a significant predictor (*p* = 0.113).

Table 7 presents an insightful thematic summary of participants’ qualitative responses concerning digital violence reporting and the institutional climate within the Faculty of Medicine. The most prominent theme is the need for an anonymous or clear reporting portal (44%). The second major theme, training and awareness through digital-safety workshops (38%). Additionally, clear university policies and guidelines (28%) emerged as a key institutional facilitator. However, confidentiality and trust concerns (21%) remain a significant barrier.

**Table 7:** Summary of qualitative themes on digital violence reporting and institutional climate among female members of the faculty of medicine (*n* = 420)

Theme	(%)	Representative Quote
Anonymous / Clear Reporting Portal	44	“If there were an anonymous reporting app, I would use it.” “A safe, anonymous online form would help.”
Training and Awareness / Digital-Safety Workshops	38	“Workshops about cyber-ethics are essential.” “We need practical short sessions.”
Confidentiality and Trust Concerns	21	“I fear my case won’t stay private.”
Clear University Policy and Guidelines	28	“Guidelines should be communicated to all staff.”

Percentages represent the proportion of participants who mentioned each theme. Since responses could include multiple themes, percentages do not sum to 100%

## Discussion

The study explores the prevalence and associated factors of digital violence within the academic environment among female members of the Faculty of Medicine at Alexandria University. It aims to identify how widespread digital violence is, the types and frequency of incidents experienced, and how individual, behavioral, and institutional factors (such as digital safety awareness, perception of cyber ethics, reporting knowledge, and barriers/facilitators) are linked to its occurrence and reporting behavior.

The results indicated a concerning level of digital violence (DV) among participants: 40% reported experiencing at least one type of online victimization. More serious forms of DV were blackmail or extortion (11.2%) and the non-consensual dissemination of intimate images (9.8%) occurred less frequently, but represent profound violations of digital ethics and privacy. With respect to frequency, nearly half (47.0%) of those affected experienced digital violence on a recurring rather than one-off basis, pointing to a pattern of repeated exposure rather than isolated incidents.

This prevalence aligns with other recent studies of technology-facilitated abuse. In a review of 74 adolescent samples from North America, Europe and Asia, authors found that cyber-dating violence victimization averaged 36.9% [21]. Meanwhile, in a European study of university students in the UK and Spain, prevalence of digital violence in intimate relationships was 15.84% in the UK and 11.05% in Spain [22, 23]. Further, in a multinational study of image-based sexual abuse (IBSA) among over 16,000 adults in 10 countries, over one in five respondents (22.6%) reported at least one incident of non-consensual image sharing or threat thereof [24].

Thus, the 40% rate observed is somewhat higher than many reported figures, underscoring the severity of sample’s exposure to digital violence.

Two-thirds of victims of DV (66.7%) reported psychological impacts, most commonly anxiety, fear and diminished self-confidence consistent with the emotional burden often associated with online harassment. Additionally, 42.3% noted that their academic or work performance suffered, indicating that digital violence can extend its harm into productivity, engagement and professional or educational functioning. This mirrors findings from cyber-victimization research showing significant associations with lower self-esteem and heightened social anxiety [25], and workplace cyber-harassment studies linking digital abuse to decreased job commitment and performance [26]. Together, the evidence suggests that the consequences of digital violence are both deeply personal and operational impairing emotional well-being as well as academic or occupational capacities.

The current study offers important insights into participants’ digital awareness, ethical sensitivity, and institutional readiness in relation to digital safety and violence-reporting. Although participants demonstrated generally positive awareness of safe digital practices and ethical perceptions, the findings also uncovered notable deficiencies in reporting knowledge, institutional support, and perceived barriers.

Overall, the moderately high mean score for digital safety awareness indicates that most individuals understand foundational elements such as secure passwords, privacy settings, and responsible information sharing. This could reflect the increasing integration of digital platforms in medical education, greater exposure to online learning environments, and heightened messaging around cyber risks via social media and institutional policies. Nonetheless, the fact that about 10% of participants scored in the low-range signals a need for targeted training interventions particularly aimed at subgroups with lower digital engagement or at higher academic ranks, where online tool use may be less frequent.

Participants’ perceptions regarding cyber ethics produced the highest mean score of all constructs studied, suggesting a strong sense of personal responsibility for online behavior. However, ethical perception alone may not translate into safe practice unless it is supported by clear institutional policies, scenario-based training, and consistent modelling of professional behavior by faculty. This supports findings that while digital ethics are being emphasized in higher education, the transition from awareness to practice remains uneven [27].

Despite satisfactory levels in awareness and ethics, the moderate mean score for reporting knowledge reveals uncertainty about formal mechanisms for reporting digital misconduct. A significant proportion of participants (60%) indicated only moderate familiarity with institutional pathways for reporting consistent with literature showing victims of online harassment often lack clarity about where and how to report [28]. This gap between awareness and action underscores the importance of developing transparent, user-friendly reporting systems, and ensuring consistent institutional communication around available channels and confidentiality protections.

Barriers to reporting remain substantial: about 30% of respondents perceived high obstacles to reporting. This finding highlighted that the mere existence of reporting procedures is insufficient without a trusting institutional culture that protects victims, ensures non-retaliatory follow-up, and demonstrates that reports lead to concrete outcomes. Research into cyber harassment in university contexts emphasizes the significance of institutional climate and visible action in determining whether individuals feel safe to come forward [28].

The moderately high score on perceived facilitators to reporting such as anonymity, digital portals, supportive staff and clear follow-up mechanisms, suggests recognition of what might encourage reporting behaviors. Training programs which emphasize step-by-step guidance on how to report, alongside visible success stories of resolved cases, could further strengthen reporting confidence and uptake.

The relatively low mean score for institutional climate or support suggests that participants perceive their institution’s environment as inconsistent or unsupportive. Strengthening the institutional climate will require more than policy drafting: enforcement, regular awareness campaigns, leadership role-modelling, and integration of digital ethics into academic governance are all critical. In fact, a recent study of higher education institutions found that the degree to which universities embed digital ethics and sustainability in curricula strongly depends on internal structures, policy commitment, and resource allocation [24, 29].

The observed difference in Digital Safety Awareness (DSA) scores between participants who had and had not experienced digital violence suggests an inverse association between awareness and vulnerability. Individuals with higher DSA levels appear more capable of recognizing and avoiding unsafe digital situations, underscoring the protective value of digital literacy. Although the effect size (Cohen’s d = 0.32) indicates a small-to-moderate relationship, the trend aligns with prior evidence that digital awareness mitigates exposure to online risks by enhancing users’ ability to manage privacy settings, detect suspicious interactions, and adopt preventive behaviors [30, 31]. Comparable findings were reported among university students in Europe and Asia, where greater cybersecurity knowledge significantly reduced the likelihood of cyber-victimization [32, 33]. This emphasizes that education on digital safety principles can serve as an essential buffer against digital harassment and exploitation. However, awareness alone may be insufficient without corresponding institutional safeguards and peer support structures [31].

The study revealed a significant negative correlation between Barriers to Reporting (BTI) and Reporting Knowledge (RK), indicating that as perceived obstacles increase, understanding of reporting procedures declines. This inverse relationship suggests that participants who are unfamiliar with formal reporting mechanisms often experience hesitation, uncertainty, or helplessness when confronted with digital harassment. Similar findings were documented among university populations in Europe and Southeast Asia, where limited knowledge of institutional processes and unclear communication channels significantly reduced reporting behavior [29, 34].

This correlation underscores the interdependence between awareness, education, and institutional trust. Effective awareness programs must therefore not only highlight online safety risks but also provide practical guidance on response and redress mechanisms. Incorporating digital reporting literacy into orientation sessions, student portals, and confidential hotlines could help lower perceived barriers and foster a culture of openness toward help-seeking [35].

Regarding help-seeking behaviors, the study highlighted a strong reliance on informal networks half of respondents (50%) preferred seeking support from friends or family rather than institutional or legal channels. This dependence may reflect low confidence in formal university systems, fear of judgment, or concerns about confidentiality. The limited proportion willing to report to supervisors (15%), university offices (10%), or leadership (6%) mirrors patterns seen in global studies, where survivors of digital violence often avoid formal channels due to anticipated inaction or reputational risk [36].

Low utilization of professional mental-health services (7%) and law enforcement (5%) further indicates that social stigma and procedural complexity remain key barriers, consistent with findings among female students in the United Kingdom and Malaysia [33, 37]. Notably, the preference for anonymous web-based forms (55%) illustrates that privacy and discretion are decisive factors influencing reporting willingness. Comparative literature confirms that anonymous and confidential reporting systems significantly enhance victim participation and trust in institutional mechanisms [28, 38].

Moderate interest in digital apps or portals (30%) and in-person offices (21%) indicates that while technology-mediated reporting is valued, some individuals still prefer direct communication if it ensures empathy and privacy. These insights suggest that institutions should implement multi-channel, survivor-centered reporting systems integrating anonymity, confidentiality, and professional support. Linking online portals with trained counselors or legal advisors may strengthen victims’ confidence and improve reporting rates [39].

Furthermore, participants’ reported interest in legal guidance (35%), case-status updates (30%), referral to counseling (28%), and the option to pause or withdraw reports (23%) reflects a need for trauma-informed, flexible frameworks that prioritize psychological safety, autonomy, and transparency. This approach is supported by trauma-informed reporting models, which stress survivor control and emotional validation throughout the reporting process [40].

The multivariate logistic regression analysis revealed that nearly half of the variance in digital violence exposure was attributable to demographic, behavioral, and psychosocial determinants. Academic role was a key predictor, with undergraduate students showing more than double the odds of experiencing digital violence compared to faculty staff. This aligns with prior studies suggesting that younger users’ greater online activity, limited digital literacy, and lower institutional authority increase vulnerability to harassment [25, 41]. Similarly, single participants were almost twice as likely to report victimization than married women, consistent with research indicating that unmarried women tend to have broader online interactions and fewer protective social boundaries [42].

Behavioral patterns also played a critical role; individuals who used the internet for more than five hours daily faced significantly higher odds of digital violence. This corroborates global findings that intensive screen time and social media engagement amplify exposure to online aggression and risky interactions [23]. Conversely, participants who had undergone digital safety training were substantially less likely to experience digital abuse supporting prior evidence that structured awareness and preventive education foster protective online behaviors [43, 44].

Psychosocial variables further illustrated resilience mechanisms. Elevated Digital Safety Awareness (DSA), Cyber-Ethics Perception (CEP), Reporting Knowledge (RK), and Facilitators of Reporting (FR) scores each exhibited protective effects, mirroring studies that emphasize the buffering role of ethical digital conduct and confidence in reporting mechanisms [45]. In contrast, high Barriers to Reporting (BTR) scores significantly increased the likelihood of victimization, underscoring how institutional inefficiency and stigma perpetuate silence and re-victimization [46]. Although Institutional Climate (IC) did not reach statistical significance (*p* = 0.113), similar findings indicate that a positive institutional ethos alone cannot mitigate risk unless reinforced by tangible enforcement, trusted channels, and transparent accountability systems [47].

Qualitative responses deepened these quantitative insights, highlighting anonymity, structured reporting systems, and awareness programs as core elements shaping reporting willingness. This echoes earlier qualitative investigations showing that survivors prioritize confidential, stigma-free mechanisms and visible institutional responsiveness when deciding to disclose digital harassment [48].

### Strengths and limitations

A major strength of this study lies in its comprehensive mixed-methods design, combining quantitative analysis with qualitative thematic validation, enhancing interpretive depth. The relatively large and diverse sample (*n* = 420) from multiple academic roles adds generalizability within medical education contexts. The use of validated, high-reliability scales (α = 0.78–0.86) also strengthens internal consistency.

However, some limitations must be acknowledged. The cross-sectional design precludes causal inference, and self-reported measures may be influenced by recall or social-desirability bias. Additionally, findings are limited to one faculty and may not generalize to other disciplines or universities. Future studies should employ longitudinal or multi-center designs and consider exploring male participants’ experiences for a broader understanding of digital safety dynamics. However, findings provide an empirical baseline for policy and educational interventions.

## Conclusions

This study shows that digital violence is a substantial and rising concern for female members of the Faculty of Medicine at Alexandria University. Nearly two out of every five participants reported experiencing some form of online harm, with many facing emotional distress and disruptions to their academic or professional performance. The results reveal that vulnerability to digital violence is shaped by a mix of demographic, behavioral, and psychosocial factors, particularly younger age, being an undergraduate or single, and spending long hours online. On the other hand, previous digital-safety training, greater awareness, and stronger cyber-ethical attitudes appeared to offer protection. Although participants displayed moderate levels of digital safety knowledge, limited understanding of reporting procedures, significant perceived barriers, and a generally weak institutional climate continue to discourage effective responses. To create a safer academic environment, universities must invest in anonymous and user-friendly reporting systems, expand digital-safety education, and implement clear, trustworthy institutional policies. A coordinated, multi-level approach is essential to empower women to seek help, reduce their risk of victimization, and strengthen digital wellbeing within medical education settings.

## Supplementary Information

Supplementary Material 1

Supplementary Material 2

Supplementary Material 3

## Data Availability

The dataset analyzed during the current study is available from the corresponding author upon reasonable request.

## Abbreviations

DV: Digital Violence
DSA: Digital Safety Awareness
CEP: Cyber–Ethics Perception
RK: Reporting Knowledge
BTR / BTI: Barriers to Reporting / Barriers to Reporting Index
FR / FTR: Facilitators of Reporting
IC: Institutional Climate
MDGs: Millennium Development Goals
GBV: Gender–Based Violence
UniSAFE: Gender–Based Violence in Research Organizations Project
