# Supplementary material for: Prevalence and correlates of digital violence among female members of the faculty of medicine, Alexandria University
Source: BMC Public Health. 2026 Apr 1;26:1173. doi: 10.1186/s12889-026-26514-1 (PMC13063572; doi:10.1186/s12889-026-26514-1)
Supplement: Supplementary file 1 — Supplementary Material 1 [file 12889_2026_26514_MOESM1_ESM.pdf]

Questionnaire: "Prevalence and Correlates of Digital Violence Among Female Members of the Faculty of Medicine, Alexandria University"

Cover Page / Participant Information & Consent
Purpose: This survey examines digital-safety awareness, cyber-ethics perceptions, experiences with digital violence, and barriers to reporting among female students, academic staff, and administrative staff at the Faculty of Medicine, Alexandria University.
Voluntary & Confidential: Participation is voluntary. You may skip any question or stop at any time. No names will be collected. Data will be reported in aggregate.
Risks/Benefits: Some questions discuss unpleasant online experiences; you may skip them. Findings will guide training, policy, and safer reporting pathways.
Time: ≈ 8–12 minutes.
Eligibility: Female (student/staff) affiliated with the Faculty of Medicine, age ≥18.
Consent:
☐ I confirm I am ≥18, female, and affiliated with the Faculty of Medicine.
☐ I have read and understood the information and agree to participate.

Definitions (read before starting):
Digital safety = practices that protect your accounts, devices, data, and privacy online.
Cyber ethics = responsible, respectful, and lawful behavior online (e.g., consent, confidentiality).
Digital violence = online or technology-facilitated behaviors that cause harm (e.g., harassment, stalking, non-consensual image sharing, impersonation, doxxing, hate speech, blackmail).

Screener (Skip Logic)
S1. Are you female and currently a student or staff member at the Faculty of Medicine, Alexandria University?

- ☐ Yes → Continue
- ☐ No → End

Section A: Socio-demographic Information
1. Age: ____ years
2. Role in Faculty: ☐ Student ☐ Academic assistant teaching ☐ Lecturer ☐ Assistant professor ☐ Professor
4. Marital status: ☐ Single ☐ Married ☐ Divorced ☐ Widowed
5. Average daily internet use: ☐ <2hours ☐ 2-5hours ☐ >5 hours
6. Primary digital platforms used (check all that apply): ☐ Facebook ☐ Instagram ☐ WhatsApp ☐ Telegram ☐ University portal ☐ Others: ___________

Section B — Digital Safety Awareness (DSA)
Scale: 1=Strongly Disagree, 2=Disagree, 3=Neutral, 4=Agree, 5=Strongly Agree
(Items marked **R** are reverse-coded)
A1. I know how to adjust privacy settings on social media.
A2. I use unique passwords for important accounts (email, banking, learning portals).
A3. I enable two-factor authentication (2FA) on key accounts.
A4. I can recognize phishing attempts (suspicious links/emails/attachments).
A5. I avoid public Wi-Fi for sensitive activities (email, banking, clinical systems).
A6. I regularly update device software/antivirus.
A7. I limit the personal information I share online.

A8. I understand secure file-sharing practices for academic/clinical materials.
A9. I can check whether a website is authentic (URL, HTTPS, certificate, spoofing cues).
A10. I know how to report an account compromise and recover access.
A11. I understand how to revoke app permissions and third-party access.
A12. I know how to securely store and back up sensitive files (e.g., research/clinical).
A13. I can identify deepfake or manipulated media cues.
A14. I know where to find official university guidance on digital safety.
A15. I sometimes reuse the same password across accounts. **(R)**

Section C — Cyber-Ethics Perception (CEP)
Scale: 1=Strongly Disagree ... 5=Strongly Agree
B1. It is unethical to share patient-related information online, even if de-identified, without formal approval.
B2. Posting identifiable photos/videos of colleagues/students without consent is unacceptable.
B3. Obtaining "consent" via pressure, status, or fear is not valid consent.
B4. Sharing non-consensual intimate images is always harmful and should be sanctioned.
B5. Cyberbullying and hate speech online are as serious as offline harassment.
B6. It is acceptable to forward "private" screenshots if it exposes wrongdoing. **(R)**
B7. Doxxing (sharing someone's private data) is never justified.
B8. Using AI tools to create deepfake images of a colleague is unethical, even as a "joke."
B9. Academic integrity online (plagiarism, data misuse) is a core ethical duty.
B10. It is okay to use another person's login for convenience if they gave it to me. **(R)**
B11. By default, I seek permission before re-sharing content that includes others.
B12. Departments should model respectful online behavior and actively discourage harassment.

Section D — Experience of Digital Violence (EDV)
Instructions: If **never experienced**, select "No" in C1 and **skip to Section D**.
C1. Have you personally experienced any digital violence in the past **12 months**?

- ☐ No → Skip to Section D
- ☐ Yes → Continue

C2. Which forms did you experience? (select all)

- ☐ Harassing or threatening messages/comments
- ☐ Cyberstalking (repeated unwanted online contact/monitoring)
- ☐ Non-consensual sharing of images (including intimate)
- ☐ Impersonation/fake accounts in my name
- ☐ Doxxing (exposure of private data, e.g., phone, address)
- ☐ Blackmail/extortion (including sexual extortion "sextortion")
- ☐ Online gender-based hate speech
- ☐ Distribution of manipulated/deepfake media
- ☐ Other: ______

C3. Where did it occur? (select all)

- ☐ WhatsApp/Telegram ☐ Facebook/Instagram ☐ X/Twitter ☐ TikTok ☐ Email
- ☐ Learning systems (e.g., LMS) ☐ Workplace tools (e.g., Teams) ☐ Gaming/Forums
- ☐ Other: ______

C4. Who was the likely perpetrator? (best guess)

- ☐ Unknown/anonymous ☐ Fellow student ☐ Patient/relative ☐ Colleague ☐ Supervisor ☐ Ex-partner/partner ☐ Other: ______

C5. Frequency in last 12 months: ☐ Once ☐ 2–3 times ☐ Monthly ☐ Weekly ☐ Daily

C6. Impact (select all): ☐ Anxiety/stress ☐ Sleep issues ☐ Social withdrawal ☐ Academic/work disruption ☐ Financial cost ☐ Safety concerns ☐ Sought medical/psychological help ☐ None
C7. Did you save evidence (screenshots, logs)? ☐ Yes ☐ No
C8. Did you report it (to platform/institution/police)? ☐ Yes (go C9) ☐ No (go C10)
C9. Where did you report? (select all)

- ☐ Platform reporting tools ☐ Faculty/University office ☐ Police/cybercrime unit ☐ Trusted person/mentor ☐ Other: ______
  Result: ☐ Resolved ☐ Partly resolved ☐ Not resolved ☐ Ongoing

C10. If you **did not report**, main reasons (select up to 3):

- ☐ Didn't know how/where to report
- ☐ Concern about confidentiality
- ☐ Fear of retaliation or reputational harm
- ☐ Belief nothing would be done
- ☐ Lack of evidence/too hard to document
- ☐ Minimized the incident/felt it was trivial
- ☐ Time/effort required
- ☐ Pressure from others not to report
- ☐ Other: ______

Section E — Reporting Knowledge & Barriers (RKB) & intention
Scale: 1=Strongly Disagree ... 5=Strongly Agree
D1. I know the formal steps to report digital harassment at the university.
D2. I know which unit/office handles digital violence complaints.
D3. I believe reports will be taken seriously.
D4. I believe reports will be handled confidentially.
D5. I believe the process is timely and fair.
D6. Fear of reputational damage discourages reporting. **(Barrier)**
D7. Fear of retaliation discourages reporting. **(Barrier)**
D8. I worry that I'll be blamed or not believed. **(Barrier)**
D9. I'm unsure what qualifies as a reportable incident. **(Barrier)**
D10. Collecting evidence is too difficult. **(Barrier)**
D11. I would be more likely to report if anonymous options were available.
D12. I would be more likely to report if I had a trained advocate supporting me.
D13. I would trust a digital reporting portal with tracking/updates.
D14. Department leaders clearly communicate zero tolerance for online abuse.
D15. I am aware of external legal options (e.g., cybercrime units).
D16. I prefer informal resolution (asking a trusted person to intervene).
D17. I would attend a short training on documentation and reporting.

Section F — Help-Seeking & Support Preferences
E1. If you experienced digital violence, who would you **most likely** contact first?

- ☐ Friend/family ☐ Class advisor/supervisor ☐ Department leadership
- ☐ University reporting office ☐ Mental-health services ☐ Police ☐ Platform tools

E2. Preferred **reporting channels** (top 3):

- ☐ Anonymous web form ☐ Named web form ☐ Email ☐ Phone hotline ☐ In-person office ☐ App/portal

E3. Which **supports** would help you report? (select all)

- ☐ Step-by-step guide ☐ Evidence documentation help ☐ Confidential advocate

- ☐ Status updates on case ☐ Option to pause or withdraw ☐ Referral to counseling ☐ Legal guidance

---

Section G — Institutional Climate & Training Needs
Scale: 1=Strongly Disagree … 5=Strongly Agree
F1. My department promotes respectful online conduct.
F2. I have seen or received clear guidance on cyber-ethics.
F3. I have seen or received clear guidance on digital safety.
F4. Staff/faculty model ethical online behavior.
F5. Students model ethical online behavior.
F6. I want short, practical training (≤60 min) on digital safety and reporting.
F7. I want specific training on identifying/managing deepfakes and impersonation.
F8. I prefer training: ☐ In-person ☐ Live online ☐ Recorded modules (select one)

Section H — Digital Habits & Exposure
G1. Primary devices (select all): ☐ Phone ☐ Laptop ☐ Tablet ☐ Shared/clinic PC
G2. Time online (avg weekday): ☐ <2h ☐ 2–4h ☐ 4–6h ☐ 6–8h ☐ >8h
G3. Main platforms used (select all): ☐ WhatsApp ☐ Facebook ☐ Instagram ☐ X ☐ TikTok ☐ YouTube ☐ LinkedIn ☐ Email ☐ LMS ☐ Other: ______
G4. Public account(s) used for academic/professional presence: ☐ Yes ☐ No
G5. Ever received unwanted contact from patients/public via social platforms? ☐ Yes ☐ No

---

Section I — Open-Ended
I1. In your own words, what would make it safer and easier to report digital violence here?
I2. What training topics in digital safety and cyber-ethics would be most useful for you?
I3. Any additional comments or suggestions?

Thank You
If this survey raised concerns and you wish to speak to someone, you may contact:

- **University support/contact:** _________________________
- **Counseling services:** _______________________________
- **Cybercrime reporting (national):** _____________________
