# Supplementary material for: Prevalence and correlates of digital violence among female members of the faculty of medicine, Alexandria University
Source: BMC Public Health. 2026 Apr 1;26:1173. doi: 10.1186/s12889-026-26514-1 (PMC13063572; doi:10.1186/s12889-026-26514-1)
Supplement: Supplementary file 2 — Supplementary Material 2 [file 12889_2026_26514_MOESM2_ESM.pdf]

Supplementary file 2: Sampling design

1) Target population & strata

Female members of the Faculty of Medicine, Alexandria University, divided into three strata:

1. Undergraduate **students**
2. Academic Faculty (teaching/clinical)
3. **Assistant teaching staff** (teaching/clinical)

Using official rosters (faculty affairs) filtered to females to build three sampling frames.

2) Primary outcome & precision

Primary proportion: prevalence of *any* digital violence in the last 12 months (binary).
Confidence level: **95% (z = 1.96)**.
Conservative prevalence for sizing: **p = 0.50** (maximizes required n).
Desired precision:

- Overall: **±5 percentage points**
- Per small strata (Assistant, faculty): **±10 percentage points** (so they're still interpretable)

Design effect (**DEFF**): 1.0 using simple random sampling inside each stratum.

Nonresponse inflation: plan +20% (adjusted if the pilot showed different).

3) Total population

- Students $N_1 = 7{,}200$
- Assistance staff $N_2 = 600$
- Academic staff $N_3 = 450$
- Total $N = 8{,}250$

4) Sample size formulas used

Cochran (initial, infinite population):

$$n_0 = \frac{z^2\, p(1-p)}{d^2}$$

Finite population correction (FPC) for overall N):

$$n = \frac{n_0}{1 + \frac{n_0 - 1}{N}}$$

Per-stratum FPC (to target per-stratum precision d_h)

$$n_h = \frac{z^2\, p(1-p)\, N_h}{d_h^2\,(N_h - 1) + z^2\, p(1-p)}$$

Nonresponse inflation: $n^{*} = \frac{n}{1-\text{nonresponse}}$

Weights for analysis: $w_h = \frac{N_h}{n_h}$

5) Worked plan (numbers you can use now)

5.1 Overall n for ±5% (for reporting the *overall* prevalence)

- $z = 1.96$, $p = 0.5$, $d = 0.05 \Rightarrow n_0 \approx 384$
- FPC with $N = 8{,}250 \rightarrow n \approx 367$
- If DEFF=1.1$\rightarrow n \approx 404$
- +20% nonresponse → $n_{\text{overall}} \approx 505$

5.2 Ensure useful precision inside small strata (admin, faculty)

Target **±10%** inside admin and faculty using the per-stratum FPC:

- Admin ($N_2 = 600$, $d_2 = 0.10$) → $n_2 \approx 83$
- Faculty ($N_3 = 450$, $d_3 = 0.10$) → $n_3 \approx 79$

Now allocate the remainder to students to keep total manageable:

- Choose **admin = 85**, **faculty = 80**
- Keep total near your 505–530 budget → set **students = 360**
- **Total** n**= 360 + 85 + 80 = 525** (still very close to what we sized and buys you precision in small strata)

5.3 What precision does this deliver?

- Students $n_1 = 360, N_1 = 7200 \rightarrow$ ME ≈ **±5.0%**
- Admin $n_2 = 85, N_2 = 600 \rightarrow$ ME ≈ **±9.8%**
- Faculty $n_3 = 80, N_3 = 450 \rightarrow$ ME ≈ **±9.9%**

(95% CIs at p=0.5, with FPC.)

5.4 Final allocation & analysis weights

- **Students:** $n_1 = 360 \rightarrow$ weight $w_1 = N_1/n_1 = 7200/360 = 20.0$
- **Admin:** $n_2 = 85 \rightarrow$ weight $w_2 = 600/85 \approx 7.06$
- **Faculty:** $n_3 = 80 \rightarrow$ weight $w_3 = 450/80 = 5.625$

Use these **base weights**; add **post-stratification** (raking) if response rates vary by year/department.

6) How to select the respondents (stratified random)

6.1 Build the frames (by stratum)

- **Students:** Female roster with program, year (1–5), department.
- **Assistant:** Female assistant list with department/clinical unit.
- **Faculty:** Female faculty list with department/clinical unit.

6.2 Draw simple random samples within each

- Assign a random number (Excel).
- Sort ascending; take the first $n_h$in each stratum.

- Keep an **oversample reserve list** (≈30% of n_h) to replace non-contacts with the **next** on the random list (no substitutions by convenience).

6.4 Contact protocol (to reduce nonresponse to ≤20%)

- Up to **3 contact attempts** at varied times/days.
- Multichannel invites (email + SMS/WhatsApp via official gateway).
- Short, mobile-friendly survey; average 8–10 min.

7) Nonresponse handling & weighting

- Track outcome codes (complete/partial/refusal/not reached/ineligible) by stratum (and by year/department).
- Compute response rates per stratum; if uneven, apply **nonresponse adjustment**: inflate weights within cells with lower response.
- Post-stratify (rake) to known margins (e.g., % by year among female students; % by department among female faculty).
