# Supplementary material for: Prevalence and correlates of digital violence among female members of the faculty of medicine, Alexandria University
Source: BMC Public Health. 2026 Apr 1;26:1173. doi: 10.1186/s12889-026-26514-1 (PMC13063572; doi:10.1186/s12889-026-26514-1)
Supplement: Supplementary file 3 — Supplementary Material 3 [file 12889_2026_26514_MOESM3_ESM.pdf]

Supplementary File 3

Psychometric Properties of Study Scales

This supplementary file presents additional psychometric properties of the study scales, including Composite Reliability (CR) values and standardized factor loadings, to support construct validity and facilitate comparison across populations. Composite reliability was calculated based on standardized factor loadings obtained from confirmatory factor analysis (CFA).

Construct	No. of Items	Cronbach's α	Composite Reliability (CR)	Standardized Factor Loadings	Interpretation
Digital Safety Awareness (DSA)	15	0.86	0.88	0.62 – 0.84	Good reliability
Cyber-Ethics Perception (CEP)	12	0.84	0.86	0.65 – 0.88	Good reliability
Reporting Knowledge (RK)	6	0.82	0.85	0.60 – 0.83	Good reliability
Barriers to Reporting (BTR)	7	0.78	0.81	0.58 – 0.79	Acceptable reliability
Facilitators of Reporting (FR)	6	0.81	0.84	0.61 – 0.85	Good reliability
Institutional Climate (IC)	5	0.80	0.83	0.63 – 0.82	Good reliability

All CR values exceeded the recommended threshold of 0.70, indicating satisfactory internal consistency. Standardized factor loadings were within acceptable ranges (>0.50), supporting convergent validity of all constructs.
